# Predictive and Reactive Distribution of Vaccines and Antivirals during Cross-Regional Pandemic Outbreaks

**DOI:** 10.1155/2011/579597

**Published:** 2011-06-05

**Authors:** Andrés Uribe-Sánchez, Alex Savachkin

**Affiliations:** Department of Industrial and Management Systems Engineering, University of South Florida, Tampa, FL 33620, USA

## Abstract

As recently pointed out by the Institute of Medicine, the existing pandemic mitigation models lack the dynamic decision support capability. We develop a large-scale simulation-driven optimization model for generating dynamic predictive distribution of vaccines and antivirals over a network of regional pandemic outbreaks. The model incorporates measures of morbidity, mortality, and social distancing, translated into the cost of lost productivity and medical expenses. The performance of the strategy is compared to that of the reactive myopic policy, using a sample outbreak in Fla, USA, with an affected population of over four millions. The comparison is implemented at different levels of vaccine and antiviral availability and administration capacity. Sensitivity analysis is performed to assess the impact of variability of some critical factors on policy performance. The model is intended to support public health policy making for effective distribution of limited mitigation resources.

## 1. Introduction

As of July 2010, WHO has reported 501 confirmed human cases of avian influenza A/(H5N1) which resulted in 287 deaths worldwide [1]. At the same time, the statistics for the H1N1 2009 outbreak has so far included 214 countries with a total reported number of infections and deaths of 419,289 and 18,239, respectively [2]. Today, an ominous expectation exists that the next pandemic will be triggered by a highly pathogenic virus, to which there is little or no pre-existing immunity in humans [3].

The nation's ability to mitigate a pandemic influenza depends on the available emergency response resources and infrastructure, and, at present, challenges abound. Predicting the exact virus subtype remains a difficult task, and even when identified, reaching an adequate vaccine supply can currently take up to nine months [4, 5]. Even if the existing vaccines prove to be potent, their availability will be limited by high production and inventory costs [6, 7] and also will be constrained by the supply of antiviral drugs, healthcare providers, hospital beds, medical supplies, and logistics. Hence, pandemic mitigation will have to be done amidst limited availability of resources and supporting infrastructure. This challenge has been acknowledged by WHO [7] and echoed by the HHS and CDC [8, 9].

The existing models on pandemic influenza (PI) containment and mitigation aims to address various complex aspects of the pandemic evolution process including: (i) the mechanism of disease progression, from the initial contact and infection transmission to the asymptomatic phase, manifestation of symptoms, and the final health outcome [10–12], (ii) the population dynamics, including individual susceptibility [13, 14] and transmissibility [10, 15–17], and behavioral factors affecting infection generation and effectiveness of interventions [18–20], (iii) the impact of pharmaceutical and nonpharmaceutical measures, including vaccination [21–23], antiviral therapy [24–26], social distancing [27–31] and travel restrictions, and the use of low-cost measures, such as face masks and hand washing [26, 32–34].

Recently, the modeling efforts have focused on combining pharmaceutical and nonpharmaceutical interventions in search for synergistic strategies, aimed at better resource utilization. Most of such approaches attempt implementing a form of social distancing followed by application of pharmaceutical measures. For significant contributions in this area see [33, 35–41]. One of the most notable among these efforts is a 2006-07 initiative by MIDAS [42], which cross-examined independent simulation models of PI spread in rural areas of Asia [43, 44], USA and UK [45, 46], and the city of Chicago [47], respectively. MIDAS cross-validated the models by simulating the city of Chicago, with 8.6M inhabitants and implementing a targeted layered containment [48, 49]. The research findings of MIDAS and some other groups [12, 33] were used in a recent “Modeling Community Containment for Pandemic Influenza” report by IOM, to formulate a set of recommendations for PI mitigation [50]. These findings were also used in a pandemic preparedness guidance developed by CDC [51].

At the same time, The IOM report [50] points out several *limitations* of the MIDAS models, observing that “because of the significant constraints placed on the models … the scope of models should be expanded.” The IOM recommends “to adapt or develop *decision-aid models* that can … provide *real-time feedback* … and include the *costs and benefits* of intervention strategies.” Our literature review yields a similar observation that most existing approaches focus on assessment of *a priori* defined strategies, and virtually none of the models are capable of *“learning,”* that is, adapting to changes in the pandemic progress, or even predicting them, to generate *dynamic* strategies. Such a strategy has the advantage of being developed dynamically, as the pandemic spreads, by selecting a mix of available mitigation options at each decision epoch, based on both the present state of the pandemic and its predicted evolution.

In an attempt to address the IOM recommendations, we present a simulation optimization model for developing predictive resource distribution over a network of regional outbreaks. The underlying simulation model mimics the disease and population dynamics of each of the affected regions (Sections 2.1 and 2.2). As the pandemic spreads from region to region, the optimization model distributes mitigation resources, including stockpiles of vaccines and antiviral and administration capacities (Section 2.3). The model seeks to minimize the impact of ongoing outbreaks and the expected impact of *potential* outbreaks, using measures of morbidity, mortality, and social distancing, translated into the cost of lost productivity and medical expenses. The methodology is calibrated and implemented on a sample outbreak in Fla, USA with over 4M inhabitants (Section 3). The strategy is compared to the reactive myopic policy, which allocates resources from one *actual* outbreak region to the next, each time trying to cover the *entire* regional population at risk, regardless of the resource availability. The comparison is done at different levels of vaccine and antiviral availability and administration capacity. We also present a sensitivity analysis for assessing the impact of variability of some critical factors, including: (i) antiviral efficacy, (ii) social distancing conformance, and (iii) CDC response delay.

## 2. Methodology

The objective of our methodology is to generate a progressive allocation of the total resource availability over a network of regional outbreaks. The methodology incorporates (i) a cross-regional simulation model, (ii) a set of single-region simulation models, and (iii) an embedded optimization model.

**Figure 1:**
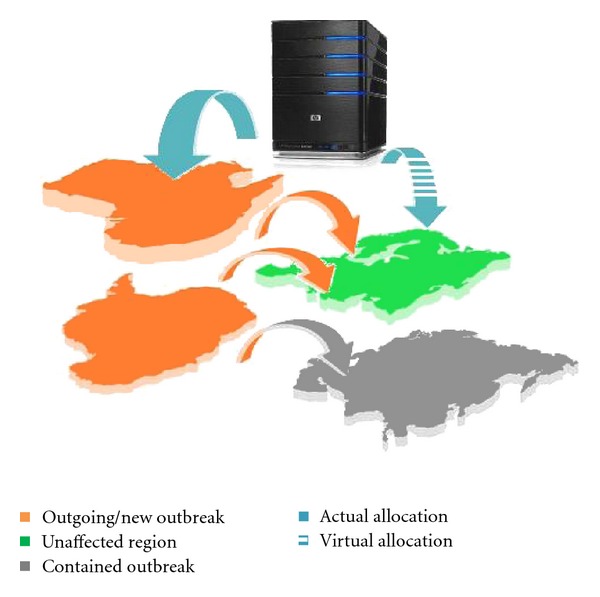
Schematic of cross-regional pandemic spread and resource distribution.

We consider a network of regions with each of which classified as either unaffected, ongoing outbreak, or contained outbreak (Figure 1). The cross-regional simulation model connects the regions by air and land travel. The single-region simulation models mimic the population and disease dynamics of each ongoing region, impacted by intervention measures. The pandemic can spread from ongoing to unaffected regions by infectious travelers who pass through regional border control. At every new regional outbreak epoch, the optimization model allocates available resources to the new outbreak region (*actual distribution*) and unaffected regions (*virtual distribution*). Daily statistics is collected for each ongoing region, including the number of infected, deceased, and quarantined cases, for different age groups. As a regional outbreak is contained, its societal and economic costs are calculated.

In Sections 2.1–2.3, we present the details of the simulation and optimization models. A testbed illustration and a comparison of our strategy to the myopic policy is given in Section 3.

### 2.1. Cross-Regional Simulation Model

**Figure 2:**
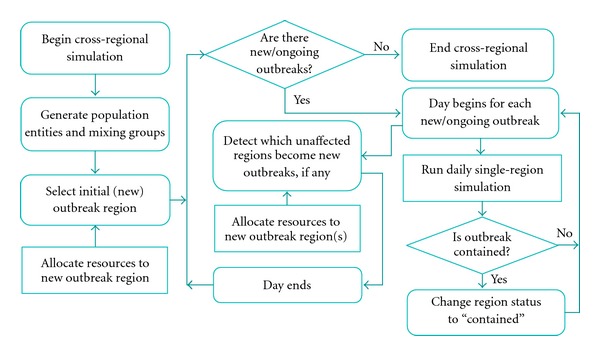
Schematic of cross-regional simulation model.

A schematic of the cross-regional simulation model is shown in Figure 2. The model is initialized by creating population entities and mixing groups, for each region. A pandemic is started by an infectious case injected into a randomly chosen region. The details of the resulting regional contact dynamics and infection transmission are given in Section 2.2. As the infected cases start seeking medical help, a new regional outbreak is detected. A resource distribution is then determined and returned to the single-region model. The outbreak can spread to unaffected regions as some infectious travelers pass undetected through the border control. By tracing these travelers, the model determines which of the unaffected regions, if any, become new outbreaks. The model also determines if any ongoing outbreaks have been contained. The simulation stops when all outbreaks are contained.

### 2.2. Single-Region Simulation Model

**Figure 3:**
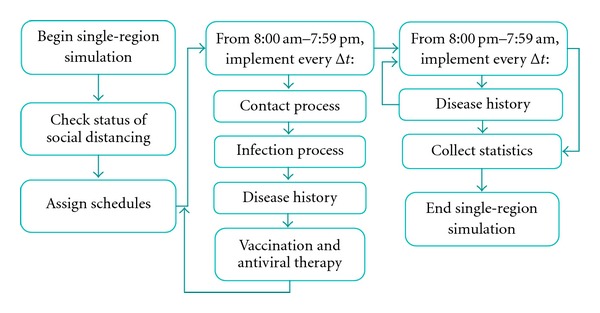
Schematic of single-region simulation model.

The single-region model subsumes the following components (see Figure 3): (i) population dynamics (mixing groups and schedules), (ii) contact and infection process, (iii) disease natural history, and (iv) mitigation strategies, including social distancing, vaccination, and antiviral application. The model collects detailed statistics, including number of infected, recovered, deceased, and quarantined cases, for different age groups. For a contained outbreak, its societal and economic costs are calculated. The societal cost includes the cost of lost lifetime productivity of the deceased; the economic cost includes the cost of medical expenses of the recovered and deceased and the cost of lost productivity of the quarantined [52].

#### 2.2.1. Mixing Groups and Schedules

Each region is modeled as a set of population centers formed by *mixing groups* or places where individuals come into contact with each other during the course of their social interaction. Examples of mixing groups include households, offices, schools, universities, shopping centers, entertainment centers, and so forth, [53]. Each individual is assigned a set of attributes such as age, gender, parenthood, workplace, infection susceptibility, and probability of travel, among others. Each person is also assigned Δ*t* time-discrete (e.g., Δ*t* = 1 hour) weekday and weekend schedules, which depend on: (i) person's age, parenthood, and employment status, (ii) disease status, (iii) travel status, and (iv) person's compliance to social distancing decrees [54]. As their schedules advance, the individuals circulate throughout the mixing groups and come into contact with each other (see Section 2.2.2).

**Figure 4:**
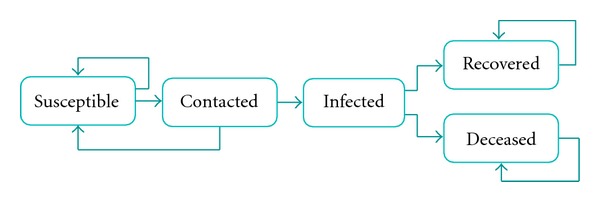
Schematic of disease natural history.

It is assumed that at any point of time, an individual belongs to one of the following compartments (see Figure 4): susceptible, contacted (by an infectious individual), infected (asymptomatic or symptomatic), and recovered/deceased. In what follows, we present the infection transmission and disease natural history model, which delineates the transitions between the above compartments.

#### 2.2.2. Contact and Infection Process

Infection transmission occurs during contact events between susceptible and infectious cases, which take place in the mixing groups. At the beginning of every Δ*t* period (e.g., one hour), for each mixing group *g*, the simulation tracks the total number of infectious cases, *n*_*g*_, present in the group. It is assumed that each infectious case generates *r*_*g*_ per Δ*t* unit of time *new contacts* [46], chosen randomly (uniformly) from the pool of susceptibles present in the group. We also assume the following: (i) during Δ*t* period, a susceptible may come into contact with at most one infectious case and (ii) each contact exposure lasts Δ*t* units of time. Once a susceptible has started her contact exposure at time *t*, she will develop infection at time *t* + Δ*t* with a certain probability that is calculated as shown below.

Let *L*_*i*_(*t*) be a nonnegative continuous random variable that represents the duration of contact exposure, starting at time *t*, required for susceptible *i* to become infected. We assume that *L*_*i*_(*t*) is distributed exponentially with mean 1/*λ*_*i*_(*t*), where *λ*_*i*_(*t*) represents the instantaneous force of infection applied to susceptible *i* at time *t* [55–57]. The probability that susceptible *i*, whose contact exposure has started at time *t*, will develop infection at time *t* + Δ*t* is then given as

(1) $$\begin{array}{c} P\left\{L_{i}\left(t\right)\leq \Delta t\right\}=1-e^{-\lambda_{i}(t)\Delta t}. \end{array}$$

#### 2.2.3. Disease Natural History

**Figure 5:**
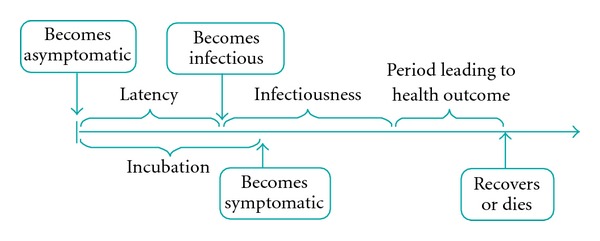
Schematic of disease natural history model.

A schematic of the disease natural history is shown in Figure 5. During the incubation phase, the infected case stays asymptomatic. At the end of the latency phase, she enters the infectious phase [44, 46, 48]. She becomes symptomatic at the end of the incubation period. At the end of the infectious phase, she enters the period leading to a health outcome, which culminates in her recovery or death.

Mortality for influenza-like diseases is a complex process affected by many factors and variables, most of which have limited accurate data support available from past pandemics. Furthermore, the time of death can sometimes be weeks following the disease episode (which is often attributable to pneumonia-related complications [58]). Because of the uncertainty underlying the mortality process, we adopted an age-based form of the mortality probability of infected *i*, as follows:

(2) $$\begin{array}{c} m_{i}=\mu_{i}\left(1-\tau \rho_{i}\right), \end{array}$$

where *μ*_*i*_ is the age-dependent base mortality probability of infected *i*, *ρ*_*i*_ is her status of antiviral therapy (0 or 1), and *τ* is the antiviral efficacy measured as the relative decrease in the base probability [44]. We assume that a recovered case develops full immunity but continues circulating in the region.

#### 2.2.4. Mitigation Strategies

Mitigation options include pharmaceutical and nonpharmaceutical interventions. Mitigation is initiated upon detection of a critical number of confirmed infected cases [59], which triggers resource distribution and deployment. The model incorporates a certain delay for deploying field responders.

*Pharmaceutical intervention (PHI)* includes vaccination and antiviral application. Vaccination is targeted at individuals *at risk* [60] to reduce their infection susceptibility. The vaccine takes a certain period to become effective [61]. Vaccination is constrained by the allocated stockpile and administration capacity, measured in terms of the immunizer-hours. We assume that as some symptomatic cases seek medical help [62, 63], those *at risk* of them will receive an antiviral. The process is constrained by the allocated stockpile and administration capacity, measured in terms of the number of certified providers.

Both vaccination and antiviral application are affected by a number of sociobehavioral factors, including conformance of the target population, degree of risk perception, and compliance of healthcare personnel [64–66]. The conformance level of the population at risk can be affected, among other factors, by the demographics and income level [67–71] as well as by the quality of public information available [54]. The degree of risk perception can be influenced by the negative experience developed during previous pharmaceutical campaigns [72, 73], as well as by public fear and rumors [74, 75].

*Nonpharmaceutical intervention (NPI)* includes social distancing and travel restrictions. We adopted a CDC guidance [51], which establishes five categories of pandemic severity and recommends quarantine and closure options according to the category. The categories are determined based on the value of the case fatality ratio (CFR), the proportion of fatalities in the total infected population. For CFR values lower than 0.1% (Category 1), voluntary at-home isolation of infected cases is implemented. For CFR values between 0.1% and 1.0% (Categories 2 and 3), in addition to at-home isolation, the following measures are *recommended*: (i) voluntary quarantine of household members of infected cases and (ii) child and adult social distancing. For CFR values exceeding 1.0% (Categories 4 and 5), all the above measures are *implemented*. As the effectiveness of social distancing is affected by some of the behavioral factors listed above [54], we assume a certain social distancing conformance level. Travel restrictions considered in the model included regional air and land border control for infected travelers.

### 2.3. Optimization Model

As presented in Figure 2, the optimization model is invoked at the beginning of every *n*th new regional outbreak epoch (*n* = 1,2,…), starting from the initial outbreak region (*n* = 1). The objective of the model is to allocate some of the available mitigation resources to the new outbreak region *(actual distribution)* while reserving the rest of the quantities for potential outbreak regions (*virtual distribution*). By doing so, the model seeks to progressively minimize the impact of ongoing outbreaks and the expected impact of potential outbreaks, spreading from the ongoing locations. Mitigation resources can include stockpiles of vaccines and antivirals, administration capacity, hospital beds, medical supplies, and social distancing enforcement resources, among others. The predictive mechanism of the optimization model is based on a set of regression equations obtained using single-region simulation models. In what follows, we present the construction of the optimization model and explain the solution algorithm for the overall simulation-based optimization methodology.

We introduce the following *general terminology and notation*:

S : Set of all network regions
A^n^ : Set of regions in which pandemic is contained at the *n*th outbreak epoch (*n* = 1,2,…)
B^n^ : Set of ongoing regions at the *n*th outbreak epoch
C^n^ : Set of unaffected regions at the *n*th outbreak epoch
R : Set of available types of mitigation resources (*R* = {1,2,…, *r*})
*q*_*ik*_ : Amount of resource *i* allocated to region *k*
*Q*_*i*_^*n*^ : Available amount of resource *i* ∈ *R* at the *n*th outbreak epoch
ℋ : Set of age groups.

*The optimization criterion* (objective function) of the model incorporates measures of expected societal and economic costs of the pandemic: the societal cost includes the cost of lost lifetime productivity of the deceased; the economic cost includes the cost of medical expenses of the recovered and deceased and the cost of lost productivity of the quarantined. To compute these costs, the following *impact measures* of morbidity, mortality, and quarantine are used, for each region *k*:

*X*_*hk*_ : Total number of infected cases in age group *h* who seek medical assistance
*Y*_*hk*_ : Total number of infected cases in age group *h* who do not seek medical assistance
*D*_*hk*_ : Total number of deceased cases in age group *h*
*V*_*hk*_ : Total number of person-days of cases in age group *h* who comply with quarantine.

To estimate these measures, we use the following regression models obtained using a single-region simulation of each region *k*:

(3) $$\begin{array}{c} X_{hk}=\delta_{hk}^{0}+\underset{i\in R}{\sum }\delta_{hk}^{i}\cdot q_{ik}+\underset{i,m\in R,\;i\neq m}{\sum }\delta_{hk}^{im}\cdot q_{ik}\cdot q_{mk}, \end{array}$$

where *δ*_··_^*i*^ denotes the regression coefficient associated with resource *i* and *δ*_··_^*im*^ is the regression coefficient for the interaction between resources *i* and *m*. Similar models are used for *Y*_*hk*_, *D*_*hk*_, and *V*_*hk*_.

The above relationships between the impact measures and the resource distributions ought to be determined *a priori* of implementing a cross-regional scenario (see Section 3). Here, we consider each region *k* as the initial outbreak region. We assume, however, that as the pandemic evolves, the disease infectivity will naturally subside. Hence, the regression equations need to be re-estimated at every new outbreak epoch, for each region *k* ∈ *C* ^*n*^, using the single-region simulation models, where each simulation must be initialized to the current outbreak status in region *k* in the cross-regional simulation. As an alternative to using a computationally burdensome approach of re-estimating the regression equations, a modeler may choose to use a certain decay factor *α* ^*n*^ [76] to adjust the estimates of the regional impact measures at every *n*th outbreak epoch, in the following way:

(4) $$\begin{array}{c} X_{hj}^{n}=\alpha^{n}X_{hj},\;\;Y_{hj}^{n}=\alpha^{n}Y_{hj},\\ D_{hj}^{n}=\alpha^{n}D_{hj},\;\;V_{hj}^{n}=\alpha^{n}V_{hj}. \end{array}$$

In addition, we use the following regression model to estimate the *probability of pandemic spread* from affected region *l* to unaffected region *k*, as a function of resources allocated to region *l*, which, in turn, impact the number of outgoing infectious travelers from the region:

(5) $$\begin{array}{c} p_{lk}=\gamma_{lk}^{0}+\underset{i\in R}{\sum }\gamma_{lk}^{i}\cdot q_{il}+\underset{\begin{array}{c} i,m\in R\\ i\neq m \end{array}}{\sum }\gamma_{lk}^{im}\cdot q_{il}\cdot q_{ml}, \end{array}$$

where *γ*_··_^*i*^ denotes the regression coefficient associated with resource *i*, *γ*_··_^*im*^ is the regression coefficient associated with interaction between resources *i* and *m*, and *γ*_··_^0^ represents the intercept. Consequently, the total outbreak probability for unaffected region *k* can be found as *p*_*k*_ = ∑_*l*∈*B*^*n*^_ *p*_*lk*_. As in the case of the impact measures, the estimates of the regional outbreak probabilities need to be progressively re-estimated or adjusted using a scheme similar to (4), as follows:

(6) $$\begin{array}{c} p_{k}^{n}=\alpha^{n}p_{k}. \end{array}$$

Finally, we calculate the total cost of an outbreak in region *k* at the *n*th decision epoch as follows:

(7) $$\begin{array}{cc} {\text{TC}}_{k}^{n} & =\underset{h\in ℋ}{\sum }\left(m_{h}+{\bar{w}}_{h}\right)X_{hk}^{n}+\underset{h\in ℋ}{\sum }{\bar{w}}_{h}\cdot Y_{hk}^{n}+\underset{h\in ℋ}{\sum }{\hat{w}}_{h}\cdot D_{hk}^{n}\\ & \;+\underset{h\in ℋ}{\sum }w_{h}\cdot V_{hk}^{n}, \end{array}$$

where *m*_*h*_ is total medical cost of an infected case in age group *h* over his/her disease period,$\;{\bar{w}}_{h}$ is total cost of lost wages of an infected case in age group *h* over his/her disease period, ${\hat{w}}_{h}$ is cost of lost lifetime wages of a deceased case in age group *h*, and *w*_*h*_ is daily cost of lost wages of a non-infected case in age group *h* who complies with quarantine.

**The model**

The optimization model has the following form.

(8) $$\begin{array}{cc} \text{Minimize} & \;{\text{TC}}_{j}^{n}\left(q_{1j},q_{2j},\ldots ,q_{rj}\right)\\ & \;+\underset{s\in C^{n}}{\sum }{\text{TC}}_{s}^{n}\left(q_{1s},q_{2s},\ldots ,q_{rs}\right)\cdot p_{s}^{n}\\ \text{subject to} & \;q_{ij}+\underset{s\in C^{n}}{\sum }q_{is}\cdot p_{s}^{n}\leq Q_{i}^{n}\;\forall i\in R,\\ & \;q_{ij},q_{is}\geq 0\;\forall i\in R. \end{array}$$

The first term of the objective function represents the total cost of the new outbreak *j*, estimated at the *n*th outbreak epoch, based on the actual resource distribution {*q*_1*j*_, *q*_2*j*_,…, *q*_*rj*_} (see (7)). The second term represents the total expected cost of outbreaks in currently unaffected regions, based on the virtual distributions {*q*_1*s*_, *q*_2*s*_,…, *q*_*rs*_} (7) and the regional outbreak probabilities *p*_*s*_^*n*^ (6). The set of constraints assures that for each resource *i*, the total quantity allocated (current and virtual, both nonnegative) does not exceed the total resource availability at the *n*th decision epoch. Note that both the objective function and the availability constraints are nonlinear in the decision variables.

### 2.4. Solution Algorithm

The solution algorithm for our dynamic predictive simulation optimization (DPO) model is given below.

(1) Estimate regression equations for each region using the single-region simulation model.
(2) Begin the cross-regional simulation model.
(3) Initialize the sets of regions: *A* ^*n*^ = *∅*, *B* ^*n*^ = *∅*, *C* ^*n*^ = *S*.
(4) Select randomly the initial outbreak region *j*. Set *n* = 1.
(5) Update sets of regions: *B* ^*n*^ ← *B* ^*n*^ ∪ {*j*} and *C* ^*n*^ ← *C* ^*n*^∖{*j*}.
(6) Solve the resource distribution model for region *j*. Update the total resource availabilities.
(7) If *B* ^*n*^ ≠ *∅*, do step 8. Else, do step 10.
(8)
  - For each ongoing region, implement a next day run of its single-region simulation.
  - Check the containment status of each ongoing region. Update sets *A* ^*n*^ and *B* ^*n*^, if needed.
  - For each unaffected region, calculate its outbreak probability.
  - Based on the outbreak probability values, determine if there is a new outbreak region(s) *j*.
  - If there is no new outbreak(s), go to step 7. Otherwise, go to step 9.
(9) For each new outbreak region *j*,
  - Increment *n* ← *n* + 1.
  - Update sets *B* ^*n*^ ← *B* ^*n*^ ∪ {*j*} and *C* ^*n*^ ← *C* ^*n*^∖{*j*}.
  - Re-estimate regression equations for each region *k* ∈ *B* ^*n*^ ∪ *C* ^*n*^ using the single-region simulations, where each simulation is initialized to the current outbreak status in the region (alternatively, use (4) and (6)).
  - Solve the resource distribution model for region *j*.
  - Update the total resource availabilities.
(10) Calculate the total cost for each contained region and update the overall pandemic cost.

## 3. Testbed Illustration

To illustrate the use of our methodology, we present a sample H5N1 outbreak scenario including four counties in Fla, USA: Hillsborough, Miami Dade, Duval, and Leon, with populations of 1.0, 2.2, 0.8, and 0.25 million people, respectively. A basic unit of time for population and disease dynamics models was taken to be Δ*t* = 1 hour. Regional simulations were run for a period (up to 180 days) until the daily infection rate approached near zero (see Section 3.3). Below, we present the details on selecting model parameter values. Most of the testbed data can be found in the supplement [77].

### 3.1. Parameter Values for Population and Disease Dynamics Models

Demographic and social dynamics data for each region [77] were extracted from the U.S. Census [78] and the National Household Travel Survey [79]. Daily (hourly) schedules [77] were adopted from [53].

Each infected person was assigned a daily travel probability of 0.24% [79], of which 7% was by air and 93% by land. The probabilities of travel among the four regions were calculated using traffic volume data [80–83], see Table 1. Infection detection probabilities for border control for symptomatic cases were assumed to be 95% and 90%, for air and land, respectively [84].

The instantaneous force of infection applied to contact *i* at time *t* ((1), [57]) was modeled as

(9) $$\begin{array}{c} \lambda_{i}\left(t\right)=-\ln\left(1-p_{i}\left(t\right)\right),\;\text{where}\;p_{i}\left(t\right)=\alpha_{i}-\delta \theta_{i}\left(t\right), \end{array}$$

where *α*_*i*_ is the age-dependent base instantaneous infection probability of contact *i*, *θ*_*i*_(*t*) is her status of vaccination at time *t* (0 or 1), and *δ* is the vaccine efficacy, measured as the reduction in the base instantaneous infection probability (achieved after 10 days [61]).

The values of age-dependent base instantaneous infection probabilities were adopted from [46] (see Table 2). The disease natural history included a latent period of 29 hours (1.21 days), an incubation period of 46 hours (1.92 days), an infectiousness period from 29 to 127 hours (1.21 to 5.29 days), and a period leading to health outcome from 127 to 240 hours (5.29 to 10 days) [85].

 Base mortality probabilities (*μ*_*i*_ in (2)) were found using the statistics recommended by the Working Group on Pandemic Preparedness and Influenza Response [52]. This data shows the percentage of mortality for age-based high-risk cases (HRC) (Table 3, columns 1–3). Mortality probabilities (column 4) were estimated under the assumption that high-risk cases are expected to account for 85% of the total number of fatalities, for each age group [52].

### 3.2. Calibration of the Single-Region Models

Single-region simulation models were calibrated using two common measures of pandemic severity [35, 45, 46]: the basic reproduction number (*R*_0_) and the infection attack rate (*IAR*). *R*_0_ is defined as the average number of secondary infections produced by a typical infected case in a totally susceptible population. *IAR* is defined as the ratio of the total number of infections over the pandemic period to the size of the initial susceptible population. To determine *R*_0_, all infected cases inside the simulation were classified by generation of infection, as in [33, 43]. The value of *R*_0_ was calculated as the average reproduction number of a typical generation in the early stage of the pandemic, with no interventions implemented (*the baseline* scenario) [33]. Historically, *R*_0_ values for PI ranged between 1.4 and 3.9 [37, 43]. To attain similar values, we calibrated the hourly contact rates of mixing groups [77] (original rates were adopted from [46]). For the four regions, the average baseline value of *R*_0_ was 2.54, which represented a high transmissibility scenario. The values of regional baseline *IAR* averaged 0.538.

### 3.3. Parameters of Mitigation

Mitigation resources included stockpiles of vaccines and antiviral and administration capacities (Section 3.4). A 24-hour delay was assumed for deployment of resources and filed responders [59].

**PHI**

The vaccination risk group included healthcare providers [66], and individuals younger than 5 years (excluding younger than 12 months old) and older than 65 years [60]. The risk group for antiviral included symptomatic individuals below 15 years and above 55 years [60, 86]. The efficacy levels for the vaccine (*δ* in (9)) and antiviral (*τ* in (2)) were assumed to be 40% [44, 87] and 70%, respectively. We did not consider the use of antiviral for a mass prophylactic reduction of infection susceptibility due to the limited antiviral availability [9] and the risk of emergence of antiviral resistant transmissible virus strains [26]. We assumed a 90% target population conformance for both vaccination and antiviral treatment [64]. The immunity development period for the vaccine was taken as 10 days [61].

**NPI**

A version of the CDC guidance for quarantine and isolation for Category 5 was implemented (Section 2.2.4, [51]). Once the reported CFR value had reached 1.0%, the following policy was declared and remained in effect for 14 days [51]: (i) individuals below a certain age *ξ* (22 years) stayed at home during the entire policy duration, (ii) of the remaining population, a certain proportion *ϕ* [88] stayed at home and was allowed a one-hour leave, every three days, to buy essential supplies, and (iii) the remaining (1 − *ϕ*) noncompliant proportion followed a regular schedule. All testbed scenarios considered the quarantine conformance level *ϕ* equal to 80% [54].

An outbreak was considered contained, if the daily infection rate did not exceed five cases, for seven consecutive days. Once contained, a region was simulated for an additional 10 days for accurate estimation of the pandemic statistics. A 2^5^ statistical design of experiment [89] was used to estimate the regression coefficient values of the significant decision factors and their interactions (see Section 2.3; the values of adjusted *R* ^2^ ranged from 96.36% to 99.97%).

The simulation code was developed using C++. The running time for a cross-regional simulation replicate involving over four million inhabitants was between 17 and 26 minutes (depending on the initial outbreak region, with a total of 150 replicates) on a Pentium 3.40 GHz with 4.0 GB of RAM.

### 3.4. Comparison of DPO and Myopic Strategies

The performance of the DPO and myopic policies is compared at different levels of resource availability.

Table 4 summarizes the total vaccine and antiviral requirements for each region, based on the composition of the regional risk groups (see Section 3.3).  Table 5 shows the per capita costs of lost productivity and medical expenses, which were adopted from [52] and adjusted for inflation for the year of 2010 [90].

**Figure 6:**
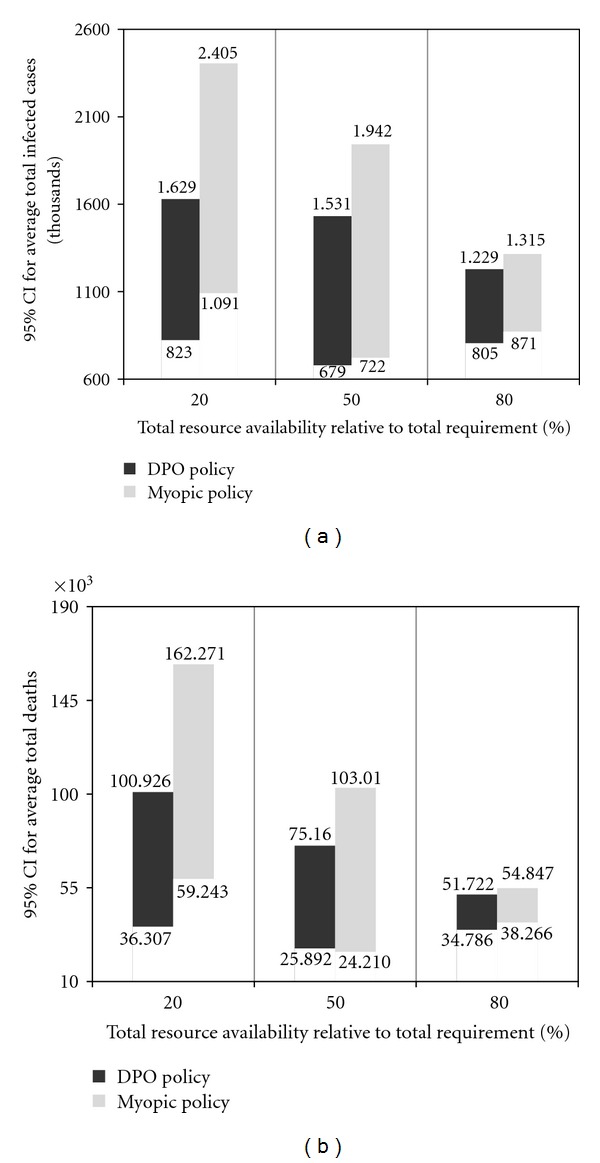
Comparison of DPO and myopic policies (average number infected 6(a) and deaths 6(b)).

 Comparison of the two strategies is done at the levels of 20%, 50%, and 80% of the total resource requirement shown in Table 4. Figures 6(a) and 6(b) show the policy comparison in the form of the 95% confidence intervals (CI) for the average number of infected and deceased, respectively. 

**Figure 7:**
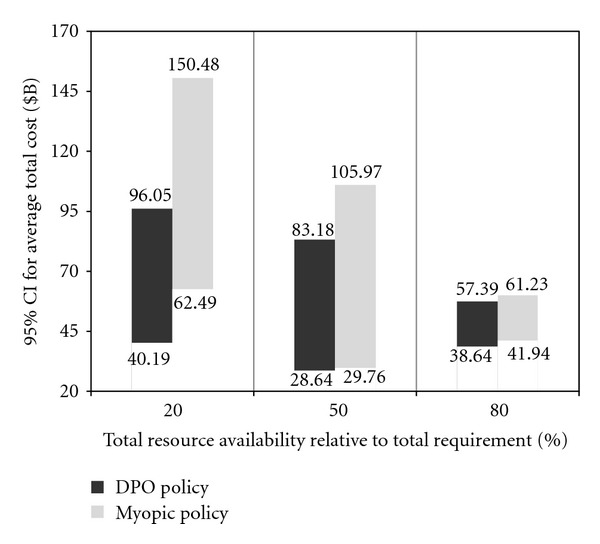
DPO versus myopic (total cost).

Figure 7 also shows the policy comparison using the 95% CI for the average total pandemic cost, calculated using the pandemic statistics, and the per capita costs from Table 5. For illustrative purposes, we also show the average number of regional outbreaks, for each policy, at different levels of resource availability, in the testbed scenario involving four regions, with the Hillsborough as the initial outbreak region (Table 6).

It can be observed that the values of all impact measures exhibit a downward trend, for both DPO and myopic policies, as the total resource availability increases from 20% to 80%.

 An increased total resource availability not only helps alleviating the pandemic impact inside the ongoing regions but also reduces the probability of spread to the unaffected regions. For both policies, as the total resource availability approaches the total resource requirement (starting from approximately 60%), the impact numbers show a converging behavior, whereby the marginal utility of additional resource availability diminishes. This behavior can be explained by noting that the total resource requirements were determined assuming the worst case scenario when *all* (four) regions would be affected and ought to provided with enough resources to cover their respective regional populations at risk. It can also be seen that on average, the DPO policy outperforms the myopic approach at all levels, which can attest to a more efficient resource utilization achieved by the DPO policy (see also Table 6). The difference in the policy performance is particularly noticeable at the lower levels of resource availability, and it gradually diminishes, as the resource availability increases and becomes closer to be sufficient to cover the entire populations at risk in all regions. It can also be noted that the variability in the performance of the DPO strategy is generally smaller than that of the myopic policy. In general, for both strategies, the performance variability decreases with higher availability of resources.

### 3.5. Sensitivity Analysis

In this section, we assess the marginal impact of variability of some of the critical factors. The impact was measured separately by the change in the total pandemic cost and the number of deaths (averaged over multiple replicates), resulting from a unit change in a decision factor value, one factor at a time. Factors under consideration included: (i) antiviral efficacy, (ii) social distancing conformance, and (iii) CDC response delay. We have used all four regions, separately, as initial outbreak regions for each type of sensitivity analysis. The results (patterns) were rather similar. Due to limited space, we have opted to show the results for only one initial region, chosen arbitrarily, for each of the three types of sensitivity studies. While Duval County was selected as the initial outbreak region to show the sensitivity results on antiviral efficacy, Hillsborough and Miami Dade were used as the initial regions to show the results on, respectively, social distancing conformance and CDC response delay. 

#### 3.5.1. Antiviral Efficacy

**Figure 8:**
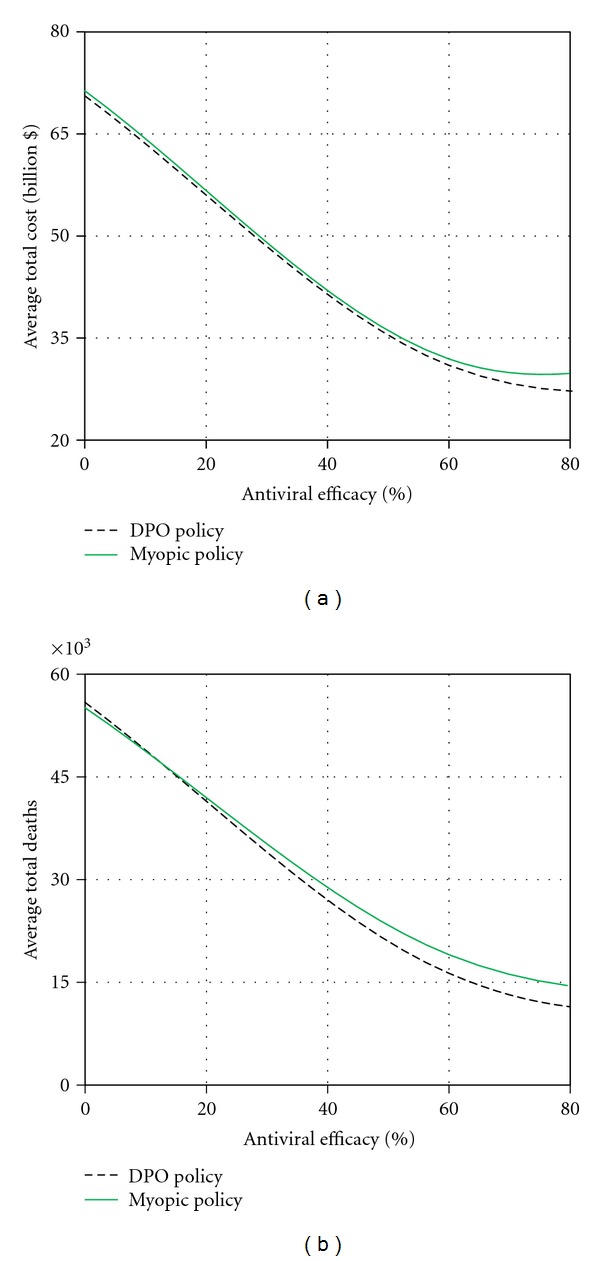
Sensitivity analysis for antiviral efficacy.

Figure 8 depicts the sensitivity of the average total cost and average total deaths to antiviral efficacy values between 0% and 80%. As expected, for both policies, the curves for the average number of deaths exhibit a decreasing trend which is almost linear for the values of *τ* between 0% and 40%. As the value of *τ* approaches 70%, the curves start exhibit a converging behavior. The curves for the average total pandemic cost exhibit a similar pattern for both policies.

It can be noted that the performance of both policies is somewhat identical for low antiviral efficacy (between 0% and 30%). However, the performance of the DPO policy improves consistently as *τ* increases which can be attributed to a more discretionary allocation of the antiviral stockpile by the DPO policy.

#### 3.5.2. Social Distancing Conformance

Reduction of the contact intensity through quarantine and social distancing has proven to be one of the most effective containment measures, especially in the early stages of the pandemic [27, 30, 31, 41].

**Figure 9:**
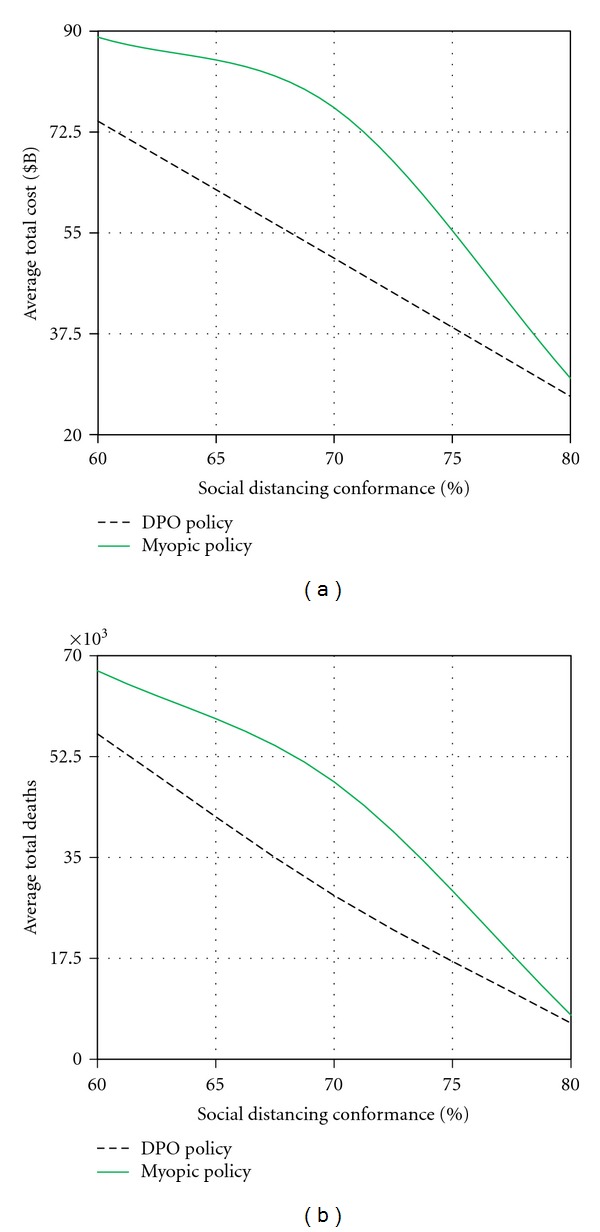
Sensitivity analysis for quarantine conformance.

Figure 9 shows the sensitivity of the average total cost and average total deaths to the social distancing conformance ranging between 60% and 80%. We observed that for both impact measures, the DPO policy demonstrated a better performance with the difference ranging from $3B to $26B in the total cost and from 1,400 to 20,000 in the number of fatalities. The biggest difference in performance was achieved at the lower-to-medium levels of conformance (between 65% and 72%). As the conformance level approached 80%, the dominating impact of social distancing masked the effect of better utilization of vaccines and antivirals achieved by the DPO strategy.

#### 3.5.3. CDC Response Delay

The CDC response delay corresponds to the interval of time from the moment an outbreak is detected to a complete deployment of mitigation resources. Depending on the disease infectivity, CDC response delay may represent one of the most critical factors in the mitigation process.

**Figure 10:**
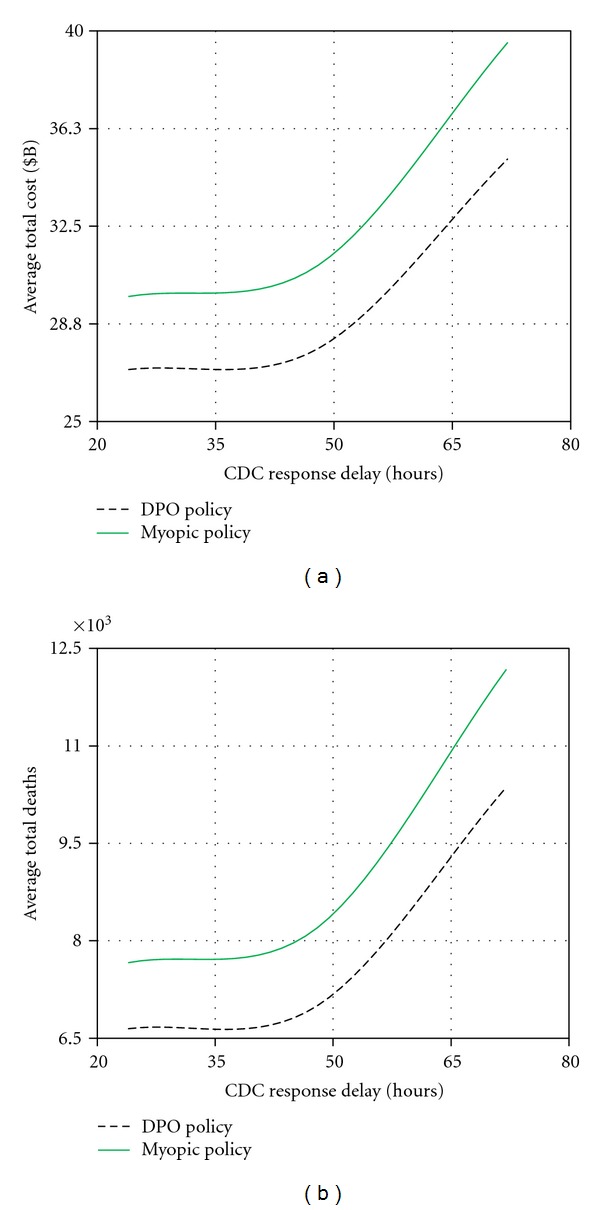
Sensitivity analysis for CDC response delay.

Figure 10 shows how the performance of both policies was significantly impacted by this factor. The DPO policy showed a uniformly better performance with the difference ranging between $3B to $4B in the average total cost, and between 800 to 1,800 in the average number of mortalities, over the range (24–72 hours) of the response delay that we examined. For both policies, there was no significant difference when the delay was between 24 and 48 hours. However, for the delay values exceeding 48 hours, the average number of deaths and total cost increased at a high rate.

## 4. Conclusions

As recently pointed by the IOM, the existing models for PI mitigation fall short of providing *dynamic* decision support which would incorporate “the costs and benefits of intervention” [50]. In this paper, we present a large-scale simulation optimization model which is attempted at filling this gap.

The model supports dynamic predictive resource distribution over a network of regions exposed to the pandemic. The model aims to balance both the ongoing and potential outbreak impact, which is measured in terms of morbidity, mortality, and social distancing, translated into the cost of lost productivity and medical expenses. The model was calibrated using historic pandemic data and compared to the myopic strategy, using a sample outbreak in Fla, USA, with over 4 million inhabitants.

*Summary of the main results*. In the testbed scenario, for both strategies, the marginal utility of additional resource availability was found to be diminishing, as the total resource availability approached the total requirement.

In the testbed scenario, the DPO strategy on average outperformed the myopic policy. As opposed to the DPO strategy, the myopic policy is reactive, rather than predictive, as it allocates resources regardless of the remaining availability and the overall cross-regional pandemic status. In contrast, the DPO model distributes resources trying to balance the impact of actual outbreaks and the expected impact of potential outbreaks. It does so by exploiting region-specific effectiveness of mitigation resources and dynamic reassessment of pandemic spread probabilities, using a set of regression submodels. Hence, we believe that in scenarios involving regions with a more heterogeneous demographics, the DPO policy will likely to perform even better and with less variability than the myopic strategy. We also note that the difference in the model performance was particularly noticeable at lower levels of resource availability, which is in accordance with a higher marginal utility of additional availability at that levels. We thus believe that the DPO model can be particularly useful in scenarios with very limited resources.

*Contributions of the paper*. The simulation optimization methodology presented in this paper is one of the first attempts to offer *dynamic predictive* decision support for pandemic mitigation, which incorporates measures of societal and economic costs. Our comparison study of the DPO versus myopic cross-regional resource distribution is also novel. Additionally, our simulation model represents one of the first of its kind in considering a broader range of social behavioral aspects, including vaccination and antiviral treatment conformance. The simulation features a flexible design which can be particularized to a broader range of PHI and NPI and even more granular mixing groups.

We also developed a decision-aid simulator which is made available to the general public through our web site at http://imse.eng.usf.edu/pandemics.aspx. The tool is intended to assist public health decision makers in implementing what-if analysis for assessment of mitigation options and development of policy guidelines. Examples of such guidelines include vaccine and antiviral risk groups, social distancing policies (e.g., thresholds for declaration/lifting and closure options), and travel restrictions.

*Limitations of the model*. Lack of reliable data prevented us from considering geo-spatial aspects of mixing group formation. We also did not consider the impact of public education and the use of personal protective measures (e.g., face masks) on transmission, again due to a lack of effectiveness data [91]. We did not study the marginal effectiveness of individual resources due to a considerable uncertainty about the transmissibility of an emerging pandemic virus and efficacy of vaccine and antiviral. For the same reason, the vaccine and antiviral risk groups considered in the testbed can be adjusted, as different prioritization schemes have been suggested. The form of social distancing implemented in the testbed can also be modified as a variety of schemes can be found in the literature, including those based on geographical and social targeting. Effectiveness of these approaches is substantially influenced by the compliance factor, for which limited accurate data support exists. It will thus be vital to gather the most detailed data on the epidemiology of a new virus and the population dynamics early in the evolution of a pandemic, and expeditiously analyze the data to adjust the interventions accordingly.

## Figures and Tables

**Table 1:** Interregional travel probabilities.

Origin*∖*Destination	Interregional Travel Probability			
	Hillsborough	Miami D.	Duval	Leon
Hillsborough	0.00	0.60	0.27	0.13
Miami D.	0.74	0.00	0.16	0.10
Duval	0.61	0.29	0.00	0.10
Leon	0.52	0.31	0.17	0.00

**Table 2:** Instantaneous infection probabilities.

Age group	0–5	6–19	20–29	31–65	66–99
*α*_*i*_	0.156	0.106	0.205	0.195	0.344

**Table 3:** Mortality probabilities for different age groups.

Age group	% HRC	% Mortality in HRC	*μ*_*i*_
0–19	6.4	9.0	0.007
20–64	14.4	40.9	0.069
65+	40.0	34.4	0.162

**Table 4:** Total and regional resource requirements.

Region (population)	Resource requirements by region				
	Hillsb.	Miami D.	Duval	Leon	Total
	(1,007,916)	(2,209,702)	(852,168)	(248,761)	(4,318,547)
Resource					
Vaccine stockpile	305,036	679,181	241,522	76,007	1,301,745
Antiviral stockpile	415,294	749,058	460,393	105,307	1,730,052
No. antiv. nurses	650	1,104	786	166	2,706
No. vacc. nurses	1,059	2,358	839	264	4,520

**Table 5:** Values of pandemic impact measures (societal and economic costs).

Pandemic impact measure (age group, years)	Value US$
Average cost of lost lifetime productivity of a deceased case (0–19)	$1,336,347.86
Average cost of lost lifetime productivity of a deceased case (20–64)	$1,370,987.28
Average cost of lost lifetime productivity of a deceased case (65–99)	$98,959.24
Average cost of lost productivity and medical expenses of a recovered/deceased case (0–19)	$5,078.48
Average cost of lost productivity and medical expenses of a recovered/deceased case (20–64)	$10,466.68
Average cost of lost productivity and medical expenses of a recovered/deceased case (65–99)	$11,566.09
Average daily cost of lost productivity of a non-infected quarantined case (20–99)	$432.54

**Table 6:** Average number of regional outbreaks for DPO and myopic policies.

	Total resource availability		
Policy	20%	50%	80%

DPO	1.75	1.66	1.44
Myopic	2.40	1.77	1.50
